# Supplementary material for: Mutant allele formation and inheritance during Cas9/guide RNA-mediated gene drive in a population modification mosquito strain for human malaria control
Source: Genetics. 2025 Aug 22;231(3):iyaf176. doi: 10.1093/genetics/iyaf176 (PMC12606424; doi:10.1093/genetics/iyaf176)
Supplement: iyaf176_Supplementary_Data [file iyaf176_supplementary_data.pdf]

Supplemental Material

Drive inheritance and mutant allele formation during Cas9/guide RNA-mediated gene drive in a population modification mosquito strain for human malaria control

Rebeca Carballar-Lejarazú, Thai Binh Pham, Taylor Tushar, Anthony A. James

Supplemental text.

Table S1. F1 progeny phenotypes and numbers from independent replicates of homozygous AgTP13 outcrosses.

Table S2. Genotypes and sequences of select F1 progeny from homozygous female AgTP13 founder outcrosses to wild-type X1 males.

Table S3. Frequently recovered MMEJ alleles.

Table S4. F2 progeny phenotypes and numbers from independent replicates of hemizygous AgTP13 testcrosses.

Table S5. Sequences of R genotypes of select F2 progeny from AgTP13 X *Agcd*$^{\Delta 11}$ testcrosses.

Table S6. Sequences of B genotypes of select F2 progeny from AgTP13 X *Agcd*$^{\Delta 11}$ testcrosses.

Table S7. Lineage-dependent sources of independently-arising mutations in the testcross F2 progeny.

Table S8. Cluster impacts on inheritance of R and B alleles in the male and female lineage testcrosses.

Figure S1 Outcross phenotype descriptions.

Figure S2. Examples of sequence tracings of cryptic and disturbed mosaic mosquitoes.

Figure S3. Distribution of R and B cluster sizes in F2 mosquitoes.

Supplemental text.

Germline and somatic amplification of mutagenic events: Similar to other Diptera, the zygote nucleus formed following fertilization in the embryo undergoes a number of mitotic divisions during a syncytial blastoderm stage (summarized in Juhn and James, 2012). Paternally- or maternally-derived Cas9/gRNA complexes in the embryo can act on the target alleles in these dividing nuclei and any resulting drive conversions or mutations will be amplified by mitotic divisions of the progeny nuclei. This increase can continue following cellularization of the nuclei to form blastoderm cells. While the majority of these cells will contribute to somatic tissues, a small number are localized to the posterior region of the embryo where they develop as pole cells and form the future diploid progenitor germline line cells. Multiple, clonally-derived converted or mutant diploid cells can manifest in an increased representation of their specific genotypes in the pole cell complement. Furthermore, the pole cells differentiate into spermatogonia in males or oogonia in females and undergo additional rounds of mitosis in the apical regions of the developing testes and ovaries, respectively (Vitale *et al*., 2023; Fiil, 1974). Here again, Cas9/gRNA-mediated activity resulting in drive or mutagenesis in one of these stem cells can be propagated clonally in the subsequent cell lineage. We know from whole-mount, *in situ* hybridization studies on these tissues that the *nanos*-driven Cas9 RNA of the core AgNosCd1 gene-drive system is present in these cells (Terradas *et al*., 2022; Carballar-Lejarazú *et al*., 2020). Further amplification is achieved in the testes when each stem cell undergoes meiosis to generate four haploid gametes. This is not seen in females as only one of the meiotic products matures as an oocyte (Fiil, 1974; Nicholson, 1921). However, as we have seen here, females can produce clusters of the same magnitude as males.

Table S1. F1 progeny phenotypes and numbers from independent replicates of homozygous AgTP13 outcrosses.

Parental cross	Rep.	CFP⁺/*cd*⁺	CFP⁺/*cd*⁻	CFP⁺/tear	CFP⁻/*cd*⁺	CFP⁻/ *cd*⁻	Tot
AgTP13 homozygous (CFP⁺/*cd*⁻; D/D) ♂ X WT-X1 (CFP⁻/*cd*⁺; W/W) ♀	1	1038	0	9	0	0	1047
	2	1008	0	9	0	0	1017
	3	1018	0	10	0	0	1028
AgTP13 homozygous (CFP⁺/*cd*⁻; D/D) ♀ X WT-X1 (CFP⁻/*cd*⁺; W/W) ♂	1	351	22	350	0	0	723
	2	161	24	353	0	0	538
	3	429	21	448	0	0	898

Abbreviations: Cyan Fluorescent Protein positive, CFP⁺; black-eye, wild-type allele of *cardinal*, *cd*⁺; red-eye, non-functional mutant allele of *cardinal*, *cd*⁻, wild-type individual, WT; wild-type allele, W; drive allele, D

Table S2. Genotypes and sequences of select F1 progeny from homozygous female AgTP13 founder outcrosses to wild-type X1 males.

Sample	Rep	Phen	Gen	Sex[1]	Mutant allele sequences[2]	Mutation type[3]
					**Reference sequence: (3'-5')**	
					CCCGAGTGGAACGGTACGGCGGTTAGCGACGATGCCAAGGCGGCCGCCATAGCGGATGGCG	
OC-929	1	CFP$^+$/*cd*$^+$	D/R	Male	CCCGAGTGGAACGGTACGGCGGTTAGCGACG------AGGCGGCCGCCATAGCGGATGGCG	-6 a
OC-973	3	CFP$^+$/*cd*$^+$	D/R	Male	CCCGAGTGGAACGGTACGGCGGTTAGCGACGATGCC---GCGGCCGCCATAGCGGATGGCG	-3
OC-978	1	CFP$^+$/*cd*$^+$	D/R	Female	CCCGAGTGGAACGGTACGGCGGTTAGC---------------GCCGCCATAGCGGATGGCG	-15
OC-1348	2	CFP$^+$/*cd*$^+$	D/R	Male	CCCGAGTGGAACGGTACGGCGGTTAGCGACGAT------GCGGCCGCCATAGCGGATGGCG	-6 b†
OC-1369	3	CFP$^+$/*cd*$^+$	D/R	Male	CCCGAGTGGAACGGTACGGCGGTTAGCGACGAT---------GCCGCCATAGCGGATGGCG	-9 c†
OC-1392	2	CFP$^+$/*cd*$^+$	D/R	Female	CCCGAGTGGAACGGTACGGCGGTTAGCGACGAT------------GCCATAGCGGATGGCG	-12 d†
OC-1411	3	CFP$^+$/*cd*$^+$	D/R	Female	CCCGAGTGGAACGGTACGGCGGTTAGCGACG------AGGCGGCCGCCATAGCGGATGGCG	-6 a
OC-1423	3	CFP$^+$/*cd*$^+$	D/R	Female	CCCGAGTGGAACGGTACGGCGGTTAGCGACGATGC-----------agccaCCATAGCGGATGGCG	-11,+5
OC-945	2	CFP$^+$/*cd*$^+$	D/R/B	Male	CCCGAGTGGAACGGTACGGCGGTTAG--------------CGGCCGCCATAGCGGATGGCG	-14†
					CCCGAGTGGAACGGTACGGCGGTTAGCGACGAT---------GCCGCCATAGCGGATGGCG	-9 c†
OC-1084	1	CFP$^+$/Tear	D/W/R	Female	CCCGAGTGGAACGGTACGGCGGTTAGCGACGAT------GCGGCCGCCATAGCGGATGGCG	-6 b†
OC-1121	3	CFP$^+$/Tear	D/W/B	Female	CCCGAGTGGAA-----------------------------CGGCCGCCATAGCGGATGGCG	-29 e†
OC-1066	3	CFP$^+$/Tear	D/R/B	Male	CCCGAGTGGAACGGTACGGCGGTT-------------AGGCGGCCGCCATAGCGGATGGCG	-13 f†
					CCCGAGTGGAACGGTACGGCGGTTAGCGACGA-----cgAGGCGGCCGCCATAGCGGATGGCG	-5,+2
OC-1091	1	CFP$^+$/Tear	D/R/B	Female	CCCGAG-------------------------------AGGCGGCCGCCATAGCGGATGGCG	-31
					CCCGAGTGGAACGGTACGGCGGTTAGCGACG-------ccatagcGGCGGCCGCCATAGCGGATGGCG	-7,+7
OC-1138	1-3	CFP$^+$/*cd*$^-$	D/B	Female	CCCGAGTGGAACGGTACGG------------------tAGGCGGCCGCCATAGCGGATGGCG	-18,+1
OC-1140	1-3	CFP$^+$/*cd*$^-$	D/B	Female	CCCGAGTGGAACGGTACGGCGGTTAGCGAC------------cctaGCCGCCATAGCGGATGGCG	-12,+4
OC-1141	1-3	CFP$^+$/*cd*$^-$	D/B	Female	CCCGAGTGGAACGGTACGGCGGTTAGCGACGAT---tagcggcggttagcgctAAGGCGGCCGCCATAGCGGATGGCG	-3,+17
OC-1144	1-3	CFP$^+$/*cd*$^-$	D/B	Female	CCCGAGTGGAACGGTACGGCGGTTAGCGACGATGCC-gccataggccgcgttAGGCGGCCGCCATAGCGGATGGCG	-1,+15
OC-1147	1-3	CFP$^+$/*cd*$^-$	D/B	Female	CCCGAGTGGAACGGTACGGCGGTTAGCGACGATGCC-gcatagcatAGGCGGCCGCCATAGCGGATGGCG	-1,+9
OC-1148	1-3	CFP$^+$/*cd*$^-$	D/B	Female	CCCGAGTGGAACGGTACGGCGGTTAGCGACGATGCC-gccatAGGCGGCCGCCATAGCGGATGGCG	-1,+5
OC-1152	1-3	CFP$^+$/*cd*$^-$	D/B	Female	CCCGAGTGGAACGGTACGGCGGTTAGCGA-----------CGGCCGCCATAGCGGATGGCG	-11 g†
OC-1153	1-3	CFP$^+$/*cd*$^-$	D/B	Female	CCCGAGTGGAACGGTACGGCGGTTAGCG---------gttAGGCGGCCGCCATAGCGGATGGCG	-9,+3 (stop)
OC-1155	1-3	CFP$^+$/*cd*$^-$	D/B	Female	CCCGAGTGGAACGGTACGGCGGTTAGCGACG-----------------ATAGCGGATGGCG	-17†
OC-1156	1-3	CFP$^+$/*cd*$^-$	D/B	Female	CCCGAGTGGAACGGTACGGCGGTTAGCGACGATGCC-AGGCGGCCGCCATAGCGGATGGCG	-1
OC-1158	1-3	CFP$^+$/*cd*$^-$	D/B	Female	CCCGAGTGGAACGGTACGGCGGTTAGCGACGATG-------------------------------ggc	-31,+3
OC-1160	1-3	CFP$^+$/*cd*$^-$	D/B	Female	CCCGAGTGGAA-----------------------------CGGCCGCCATAGCGGATGGCG	-29 e†
OC-1161	1-3	CFP$^+$/*cd*$^-$	D/B	Female	CCCGAGTGGAACGGTAC---------------------GGCGGCCGCCATAGCGGATGGCG	-21 h†

OC-1163	1-3	CFP$^+$/*cd*$^-$	D/B	Male	CCCGAGTGGAACGGTACGGCGGTT-------------AGGCGGCCGCCATAGCGGATGGCG	-13 f†
OC-1165	1-3	CFP$^+$/*cd*$^-$	D/B	Male	CCCGAGTGGAACGGTACGGCGGTTAGCGACGATGC--gttagcgAGGCGGCCGCCATAGCGGATGGCG	-2,+7
OC-1166	1-3	CFP$^+$/*cd*$^-$	D/B	Male	CCCGAGTGGAACGGTACGGCGGTTAGCGA-------AAGGCGGCCGCCATAGCGGATGGCG	-7
OC-1167	1-3	CFP$^+$/*cd*$^-$	D/B	Male	CCCGAGTGGAACGGTACGGCGGTTAGCGACGA-------ccataGCGGCCGCCATAGCGGATGGCG	-7,+5
OC-1172	1-3	CFP$^+$/*cd*$^-$	D/B	Male	CCCGAGTGGAACGGTACGGCGGTT-------------AGGCGGCCGCCATAGCGGATGGCG	-13 f†
OC-1157	1-3	CFP$^+$/*cd*$^-$	D/B	Female	CCCGAGTGGAACGGTACGGCGGTTAGCGACGATGCCA------------tgtTAGCGGATGGCG	-12,+3
OC-1139	1-3	CFP$^+$/*cd*$^-$	D/B/B	Female	CCCGAGTGGAACGGTACGGCGGTTAGCGACGATG-------------cggCATAGCGGATGGCG	-13,+3
					CCCGAGTGGAACGGTACGGCGGTTAGCGACGATGCCAtagcgccgccatAGGCGGCCGCCATAGCGGATGGCG	+12 (stop)
OC-1146	1-3	CFP$^+$/*cd*$^-$	D/B/B	Female	CCCGAGTGGAACGGTAC--	-49
					CCCGAGTGGAACGGTAC---------------------GGCGGCCGCCATAGCGGATGGCG	-21 h†
OC-1149	1-3	CFP$^+$/*cd*$^-$	D/B/B	Female	CCCGAGTGGAACGGT------------------------GCGGCCGCCATAGCGGATGGCG	-24
					CCCGAGTGGAACGGTACGGCGGTTAGCGACGATG--------------gATAGCGGATGGCG	-14,+1
OC-1154	1-3	CFP$^+$/*cd*$^-$	D/R/B	Female	CCCGAGTGGAACGGTACGGCGGTTAGCGACGAT------------GCCATAGCGGATGGCG	-12 d†
					CCCGAGTGGAACGGTACGGCGGTTAGCGACGATGCC-gcgatagatAGGCGGCCGCCATAGCGGATGGCG	-1,+9
OC-1159	1-3	CFP$^+$/*cd*$^-$	D/B/B	Female	CCCGAGTGGAACGGT------------------------------------GCGGATGGCG	-36
					CCCGAGTGGAACGGTACGGCGGTTAGCGACGATGCCAcgatgccttAGGCGGCCGCCATAGCGGATGGCG	+9 (stop)
OC-1162	1-3	CFP$^+$/*cd*$^-$	D/B/B	Female	CCCGAGTGGAACGGTACGGCGGTTAGCGACGATGC-----CGGCCGCCATAGCGGATGGCG	-5†
					CCCGAGTGGAACGGTACGGCGGTT-------------AGGCGGCCGCCATAGCGGATGGCG	-13 f†
OC-1168	1-3	CFP$^+$/*cd*$^-$	D/B/B	Male	CCCGAGTGGAA-------------------------------------ggccgctATAGCGGATGGCG	-37,+7
					CCCGAGTGGAACGGTACGGCGGTTAGCGAC--------------cgccatggaggccggCGCCATAGCGGATGGCG	-14,+15
OC-1169	1-3	CFP$^+$/*cd*$^-$	D/B/B	Male	CCCGAGTGGAACGGTACGGCGGTTAGCGAC-------------------------------	-83
					CCCGAGTGGAACGGTACGGCGGTTAGCGACGATGCCAAtcggatcGGCGGCCGCCATAGCGGATGGCG	+7
OC-1170	1-3	CFP$^+$/*cd*$^-$	D/B/B	Male	CCCGAGTGGAACGGT-------------------------------CCATAGCGGATGGCG	-31
					CCCGAGTGGAACGGTACGGCGGTTAGCGA-----------CGGCCGCCATAGCGGATGGCG	-11 g†

[1]Sex refers to the sex of the mosquitoes and how they were used in the subsequent testcrosses prior to the sequencing of their DNA.
[2]Wild-type sequences are not listed.
[3]Mutation type: negative numbers are deletions; positive numbers are insertions; a-h are identical genotypes in multiple samples of outcross progeny; † are sequences with microhomology end-joining features of 2-5-base-pair direct repeats in the canonical wild-type sequence.
Underlining indicates direct or inverted sequence insertions identical to a nearby genomic sequence.
Green shading highlights genotypes that also were found in subsequent testcrosses samples (Tables S5 and S6).
Abbreviations: Rep, replicate number; Phen, phenotype; Gen, genotype; CFP$^+$ cyan fluorescent protein positive; *cd*$^-$, mutant *cardinal* gene; *cd*$^+$, wild-type *cardinal* gene; D, drive allele; R, functional mutant allele; B, non-functional mutant allele; W, wild-type allele. Sequence font colors represent the gRNA target, the PAM site and a deletion or insertion.

**Table S3. Frequently recovered MMEJ deletion alleles.**	
**Wild-type *Agcd***	CCCGAGTGGAACGGTACGGCGGTTAGCGACGATGCCAAGGCGGCCGCCATAGCGGATGGCG
**Mutation: -11 bp**<br>**AcTP43 maternal effect[4]**<br>**AgNosCd-1 cage trial[5]**<br>**AgNosCd-1 maternal effect[6]**<br>**AgNosCd1 in *An. gambiae* NDokayo[7]**<br>**AgTP13/*Agcd*$^{\Delta 11}$ testcross[8]**	CCCGAGTGGAACGGTACGGCGGTTAGCGACGGCCGCCATAGCGGATGGCG[1]<br>CCCGAGTGGAACGGTACGGCGGTTAGCGA-----------<u>CG</u>GCCGCCATAGCGGATGGCG[2]<br>CCCGAGTGGAACGGTACGGCGGTTAGCGA<u>CG</u>-----------GCCGCCATAGCGGATGGCG[3]
**Mutation: -13 bp**<br>**AcTP43 maternal-effect[4]**<br>**AgNosCd-1 in *An. coluzzii* Mopti[7]**<br>**AgTP13 hemi female outcross[8]**<br>**AgTP13/*Agcd*$^{\Delta 11}$ testcross[8]**<br>**AgTP13 cage trial[9]**	CCCGAGTGGAACGGTACGGCGGTTAGGCGGCCGCCATAGCGGATGGCG[1]<br>CCCGAGTGGAACGGTACGGCGGTT-------------<u>AG</u>GCGGCCGCCATAGCGGATGGCG[2]<br>CCCGAGTGGAACGGTACGGCGGTT<u>AG</u>-------------GCGGCCGCCATAGCGGATGGCG[3]
**Mutation: -14 bp**<br>**AgNosCd-1 cage trial[5]**<br>**AgNosCd-1 in Kisumu[7]**<br>**AcTP13 hemi female outcross[8]**<br>**AgTP13/*Agcd*$^{\Delta 11}$ testcross[8]**	CCCGAGTGGAACGGTACGGCGGTTAGCGGCCGCCATAGCGGATGGCG[1]<br>CCCGAGTGGAACGGTACGGCGGTTA<u>GCG</u>--------------GCCGCCATAGCGGATGGCG[2]<br>CCCGAGTGGAACGGTACGGCGGTTA--------------<u>GCG</u>GCCGCCATAGCGGATGGCG[3]

[1]Recovered sequence from Sanger sequencing before alignment.
[2]Alignment output using Snapgene.
[3]Alternative alignment showing microhomology (underlined).
[4]Unpublished.
[5]Carballar *et al.*, 2020.
[6]Carballar *et al.*, 2022.
[7]Tushar *et al.*, 2024.
[8]This study.
[9]Carballar *et al.*, 2023.
Abbreviation; bp, base-pairs; hemizygous, hemi.
Sequence font colors represent the gRNA target, the PAM site and dashes are the deletion.

Table S4. F2 progeny phenotypes and numbers from independent replicates of hemizygous AgTP13 testcrosses.

Parental cross	Rep.	CFP$^+$/*cd*$^+$	CFP$^+$/*cd*$^-$	CFP$^+$/tear	CFP$^-$/*cd*$^+$	CFP$^-$/ *cd*$^-$	Total
**Male lineage**							
**F1 CFP$^+$/*cd*$^+$ ♂ X *Agcd*$^{\Delta 11}$ ♀**	1	0	1291	0	0	1	1292
	2	0	1002	0	0	0	1002
	3	0	1193	0	4	0	1197
**F1 CFP$^+$/*cd*$^+$ ♀ X *Agcd*$^{\Delta 11}$ ♂**	1	0	1072	0	44	3	1119
	2	0	848	0	15	0	863
	3	0	1010	0	32	2	1044
**F1 CFP$^+$/tear ♂ X *Agcd*$^{\Delta 11}$ ♀**	Combined F1 parents from Reps 1-3	0	230	0	0	3	233
**F1 CFP$^+$/tear ♀ X *Agcd*$^{\Delta 11}$ ♂**	Combined F1 parents from Reps 1-3	0	384	0	3	1	388
**Female lineage**							
**F1 CFP$^+$/*cd*$^+$ ♂ X *Agcd*$^{\Delta 11}$ ♀**	1	0	576	0	41	0	617
	2	0	503	0	18	30	551
	3	0	711	0	22	0	733
**F1 CFP$^+$/*cd*$^+$ ♀ X *Agcd*$^{\Delta 11}$ ♂**	1	0	720	0	62	0	782
	2	0	704	0	34	6	744
	3	0	462	0	43	33	538
**F1 CFP$^+$/tear ♂ X *Agcd*$^{\Delta 11}$ ♀**	1	0	468	0	0	11	479
	2	0	451	0	1	6	458
	3	0	701	0	5	10	716
**F1 CFP$^+$/tear ♀ X *Agcd*$^{\Delta 11}$ ♂**	1	0	610	0	32	8	650
	2	0	678	0	9	1	688
	3	0	723	0	17	15	745
**F1 CFP$^+$/*cd*$^-$ ♂ X *Agcd*$^{\Delta 11}$ ♀**	Combined F1 parents from Reps 1-3	0	167	0	0	163	330
**F1 CFP$^+$/*cd*$^-$ ♀ X *Agcd*$^{\Delta 11}$ ♂**	Combined F1 parents from Reps 1-3	0	276	0	29	219	524

Abbreviations: Cyan Fluorescent Protein positive, CFP$^+$; black-eye, wild-type allele of *cardinal*, *cd*$^+$; red-eye, non-functional mutant allele of *cardinal*, *cd*$^-$.

Table S5. Sequences of R genotypes of select F2 progeny from AgTP13 X *Agcd*$^{\Delta 11}$ testcrosses.

Parental cross	Sample	Rep	Phen	Gen	Reference sequence: (3'-5')<br>CCCGAGTGGAACGGTACGGCGGTTAGCGACGATGCCAAGGCGGCCGCCATAGCGGATGGCG<br><br>Mutant allele sequences[1, 2]	Mutation type[3]
**F1 parents from homozygous AgTP13 male outcrosses**						
**F1 CFP^{+}/*cd*$^{+}$ ♂ X *Agcd*$^{\Delta 11}$ ♀**	TC-661 **(OC-1084 and OC-1348)**	3	**CFP^{-}/*cd*$^{+}$**	**R/*cd*$^{\Delta 11}$**	CCCGAGTGGAACGGTACGGCGGTTAGCGACGAT------GCGGCCGCCATAGCGGATGGCG (n=1)	-6 b†
**F1 parents from homozygous AgTP13 female outcrosses**						
**F1 CFP^{+}/*cd*$^{+}$ ♂ X *Agcd*$^{\Delta 11}$ ♀**	TC-281-300 (**OC-945 and** OC-1369)	1	**CFP^{-}/*cd*$^{+}$**	**R/*cd*$^{\Delta 11}$**	CCCGAGTGGAACGGTACGGCGGTTAGCGACGAT---------GCCGCCATAGCGGATGGCG (n=20)	-9 c†
	TC-301-317 **(OC-945 and** OC-1369)	2	**CFP^{-}/*cd*$^{+}$**	**R/*cd*$^{\Delta 11}$**	CCCGAGTGGAACGGTACGGCGGTTAGCGACGAT---------GCCGCCATAGCGGATGGCG (n=17)	-9 c†
	TC-318-339 (**OC-929 and** OC-1411)	3	**CFP^{-}/*cd*$^{+}$**	**R/*cd*$^{\Delta 11}$**	CCCGAGTGGAACGGTACGGCGGTTAGCGACG------AGGCGGCCGCCATAGCGGATGGCG (n=22)	-6 a
**F1 CFP^{+}/*cd*$^{+}$ ♀ X *Agcd*$^{\Delta 11}$ ♂**	TC-349, 351 **(OC-978)**	1	**CFP^{-}/*cd*$^{+}$**	**R/*cd*$^{\Delta 11}$**	CCCGAGTGGAACGGTACGGCGGTTAGC---------------GCCGCCATAGCGGATGGCG (n=2)	-15†
	TC-362, 365, 366, 371, 373, 377-380, 647-660 **(OC-1154 and OC-1392)**	2	**CFP^{-}/*cd*$^{+}$**	**R/*cd*$^{\Delta 11}$**	CCCGAGTGGAACGGTACGGCGGTTAGCGACGAT------------GCCATAGCGGATGGCG (n=23)	-12†
	TC-382, 383, 387, 388, 390, 392, 395, 399 **(OC-929 and OC-1411)**	3	**CFP^{-}/*cd*$^{+}$**	**R/*cd*$^{\Delta 11}$**	CCCGAGTGGAACGGTACGGCGGTTAGCGACG------AGGCGGCCGCCATAGCGGATGGCG (n=8)	-6 a
**F1 CFP^{+}/Tear ♂ X *Agcd*$^{\Delta 11}$ ♀**	TC-421	2	**CFP^{-}/*cd*$^{+}$**	**R/*cd*$^{\Delta 11}$**	CCCGAGTGGAACGGTACGGCGGTTAGCGACGATG-aCAAGGCGGCCGCCATAGCGGATGGCG (n=1)	-1,+1
	TC-422-425	3	**CFP^{-}/*cd*$^{+}$**	**R/*cd*$^{\Delta 11}$**	CCCGAGTGGAACGGTACGGCGGTTAGCGACGATGCC---gatGCGGCCGCCATAGCGGATGGCG (n=4)	-3,+3
**F1 CFP^{+}/ Tear ♀ X *Agcd*$^{\Delta 11}$ ♂**	TC-426, 428, 432, 433, 436, 438, 440, 441, 444, 445, 450, 451, 453, 454 **(OC-1084)** and **OC-1348)**	1	**CFP^{-}/*cd*$^{+}$**	**R/*cd*$^{\Delta 11}$**	CCCGAGTGGAACGGTACGGCGGTTAGCGACGAT------GCGGCCGCCATAGCGGATGGCG (n=14)	-6 b†
	TC-427, 429, 430, 442, 443, 446, 447, 448, 452 **(OC-929 and OC-1411)**	1	**CFP^{-}/*cd*$^{+}$**	**R/*cd*$^{\Delta 11}$**	CCCGAGTGGAACGGTACGGCGGTTAGCGACG------AGGCGGCCGCCATAGCGGATGGCG (n=9)	-6 a
	TC-463, 464, 466, 467, 468, 470, 476, 477, 644-646 **(OC-945 and OC-1369)**	3	**CFP^{-}/*cd*$^{+}$**	**R/*cd*$^{\Delta 11}$**	CCCGAGTGGAACGGTACGGCGGTTAGCGACGAT---------GCCGCCATAGCGGATGGCG (n=11)	-9 c†
	TC-471, 473 **(OC-929 and OC-1411)**	3	**CFP-/cd+**	**R/*cd*$^{\Delta 11}$**	CCCGAGTGGAACGGTACGGCGGTTAGCGACG------AGGCGGCCGCCATAGCGGATGGCG (n=2)	-6 a
**F1 CFP^{+}/*cd*$^{-}$ ♀ X *Agcd*$^{\Delta 11}$ ♂**	TC-401, 403, 404, 405, 407, 409, 410, 411, 412, 414, 419, 420	1-3	**CFP^{-}/*cd*$^{+}$**	**R/*cd*$^{\Delta 11}$**	CCCGAGTGGAACGGTACGGCGGTTAGCGAC---------GCGGCCGCCATAGCGGATGGCG (n=12)	-9†

	TC-402, 413, 415-418	1-3	**CFP$^-$/*cd*$^+$**	**R/*cd*$^{\Delta 11}$**	CCCGAGTGGAACGGTACGGCGGTTAGC---------------tacgatGCCGCCATAGCGGATGGCG (n=6)	-15,+6
	TC-406	1-3	**CFP$^-$/*cd*$^+$**	**R/*cd*$^{\Delta 11}$**	CCCGAGTGGAACGGTACGGCGGTTAGCGACGATGCCA--------taGCCATAGCGGATGGCG (n=1)	-8,+2

[1]Wild-type and *cd*$^{\Delta 11}$ sequences are not listed.
[2]n is the number of that genotype sequenced.
[3]Mutation type: negative numbers are deletions; positive numbers are insertions; a-c are identical genotypes in multiple samples of F2 testcross progeny with letters from the F1 outcross progeny; † are sequences with microhomology end-joining features of 2-5-base-pair direct repeats in the canonical wild-type sequence.
Green shading highlights genotypes that also were found in outcross samples (OC sample identity in bold in parentheses).
Magenta highlighting signifies a possible source of an inherited allele from an OC individual (Table S2).
Grey highlighting signifies an inferred inherited allele based on the specific cross.
Abbreviations: Rep, replicate number; Phen, phenotype; Gen, genotype; CFP$^+$ cyan fluorescent protein positive; *cd*$^-$, mutant *cardinal* gene; *cd*$^+$, wild-type *cardinal* gene; D, drive allele; R, functional mutant allele; B, non-functional mutant allele; W, wild-type allele. Sequence font colors represent the gRNA target, the PAM site and deletion or insertion.

Table S6. Sequences of B genotypes of select F2 progeny from AgTP13 X *Agcd*$^{\Delta11}$ testcrosses.

Parental cross	Sample	Rep	Phen	Gen	Reference sequence: (3'-5')<br>CCCGAGTGGAACGGTACGGCGGTTAGCGACGATGCCAAGGCGGCCGCCATAGCGGATGGCG<br><br>Independently-derived mutant alleles[1,2]	Mutation type[3]
**F1 parents from homozygous AgTP13 male outcrosses**						
**F1 CFP^{+}/*cd*$^{+}$ ♂ X *Agcd*$^{\Delta11}$ ♀**	TC-746 **(OC-1152 and OC-1170)**	1	**CFP^{-}/*cd*$^{-}$**	**B/*cd*$^{\Delta11}$**	CCCGAGTGGAACGGTACGGCGGTTAGCGA-----------CGGCCGCCATAGCGGATGGCG (n=1)	-11 g†
**F1 CFP^{+}/*cd*$^{+}$ ♀ X *Agcd*$^{\Delta11}$ ♂**	TC-747-749 **(OC-1156)**	1	**CFP^{-}/*cd*$^{-}$**	**B/*cd*$^{\Delta11}$**	CCCGAGTGGAACGGTACGGCGGTTAGCGACGATGCC-AGGCGGCCGCCATAGCGGATGGCG (n=3)	-1 i
	TC-750-751	3	**CFP^{-}/*cd*$^{-}$**	**B/*cd*$^{\Delta11}$**	CCCGAGTGGAACGGTACGGCGGTTAGCGAC-------AGGCGGCCGCCATAGCGGATGGCG (n=2)	-7
**F1 CFP^{+}/Tear ♂ X *Agcd*$^{\Delta11}$ ♀**	TC-755-757	1-3	**CFP^{-}/*cd*$^{-}$**	**B/*cd*$^{\Delta11}$**	CCCGAGTGGAACGGTACGGCGGT----------------GCGGCCGCCATAGCGGATGGCG (n=3)	-16
**F1 parents from homozygous AgTP13 female outcrosses**						
**F1 CFP^{+}/*cd*$^{+}$ ♂ X *Agcd*$^{\Delta11}$ ♀**	TC-479-481, 483-498, 500-505 **(OC-945 and OC-1369)**	2	**CFP^{-}/*cd*$^{-}$**	**B/*cd*$^{\Delta11}$**	CCCGAGTGGAACGGTACGGCGGTTAG--------------CGGCCGCCATAGCGGATGGCG (n=25)	-14 j†
	TC-482, 499	2	**CFP^{-}/*cd*$^{-}$**	**B/*cd*$^{\Delta11}$**	CCCGAGTGGAACGGTACGGCGGTTAGCGACGAT----------CCGCCATAGCGGATGGCG (n=2)	-10
**F1 CFP^{+}/*cd*$^{+}$ ♀ X *Agcd*$^{\Delta11}$ ♂**	TC-506-510 **(OC-1156)**	2	**CFP^{-}/*cd*$^{-}$**	**B/*cd*$^{\Delta11}$**	CCCGAGTGGAACGGTACGGCGGTTAGCGACGATGCC-AGGCGGCCGCCATAGCGGATGGCG (n=5)	-1 i
	TC-511-538	3	**CFP^{-}/*cd*$^{-}$**	**B/*cd*$^{\Delta11}$**	CCCGAGTGGAACGGTACGGCGGTTAGCGACGATG--<br>gctatggcggttgAGGCGGCCGCCATAGCGGATGGCG (n=28)	-2,+13 k
**F1 CFP^{+}/Tear ♂ X *Agcd*$^{\Delta11}$ ♀**	TC-601, 609, 611	1	**CFP^{-}/*cd*$^{-}$**	**B/*cd*$^{\Delta11}$**	CCCGAGTGGAACGGTACGGCGGTTAGC---------------------cggactaATAGCGGATGGCG (n=7)	-21,+7
	TC-610 **(OC-1156)**	1	**CFP^{-}/*cd*$^{-}$**	**B/*cd*$^{\Delta11}$**	CCCGAGTGGAACGGTACGGCGGTTAGCGACGATGCC-AGGCGGCCGCCATAGCGGATGGCG (n=1)	-1 i
	TC-599-603	2	**CFP^{-}/*cd*$^{-}$**	**B/*cd*$^{\Delta11}$**	CCCGAGTGGAACGGTACGGCGGTTAGCGACGATGCC---gcGCGGCCGCCATAGCGGATGGCG (n=5)	-3,+2
	TC-612, 613, 614, 618, 619, 620 **(OC-945)**	3	**CFP^{-}/*cd*$^{-}$**	**B/*cd*$^{\Delta11}$**	CCCGAGTGGAACGGTACGGCGGTTAG--------------CGGCCGCCATAGCGGATGGCG (n=6)	-14 j†
	TC-615-617	3	**CFP^{-}/*cd*$^{-}$**	**B/*cd*$^{\Delta11}$**	CCCGAGTGGAACGGTACGGCGGTTAGCGACG---------<br>ccccgccgtcatagCGGCCGCCATAGCGGATGGCG (n=3)	-9,+14
**F1 CFP^{+}/ Tear ♀ X *Agcd*$^{\Delta11}$ ♂**	TC-621-628	1	**CFP^{-}/*cd*$^{-}$**	**B/*cd*$^{\Delta11}$**	CCCGAGTGGAACGGTACGGCGGTTAGCGACG-------------------AGCGGATGGCG (n=8)	-19†
	TC-629 **(OC-1152 and OC-1170)**	2	**CFP^{-}/*cd*$^{-}$**	**B/*cd*$^{\Delta11}$**	CCCGAGTGGAACGGTACGGCGGTTAGCGA-----------CGGCCGCCATAGCGGATGGCG (n=1)	-11 g†
	TC-630, 631, 633, 637, 638 **(OC-1121** and **OC-1160)**	3	**CFP^{-}/*cd*$^{-}$**	**B/*cd*$^{\Delta11}$**	CCCGAGTGGAA-----------------------------CGGCCGCCATAGCGGATGGCG (n=5)	-29†
	TC-632, 635, 639, 641, 642	3	**CFP^{-}/*cd*$^{-}$**	**B/*cd*$^{\Delta11}$**	CCCGAGTGGAACGGTACGGCGGTTAGCGACGATGCCAtAGGCGGCCGCCATAGCGGATGGCG (n=5)	+1
	TC-634, 636, 640, 643 **(OC-1152 and OC-1170)**	3	**CFP^{-}/*cd*$^{-}$**	**B/*cd*$^{\Delta11}$**	CCCGAGTGGAACGGTACGGCGGTTAGCGA-----------CGGCCGCCATAGCGGATGGCG (n=4)	-11 g†

**F1 CFP^{+}/*cd*$^{-}$ ♂ X *Agcd*$^{\Delta 11}$ ♀**	TC-539, 548	1-3	**CFP^{-}/*cd*$^{-}$**	**B/B/*cd*$^{\Delta 11}$**	CCCGAGTGGAACGGTACGGCGGTTAGCGACGA-----------------cgcctgtTAGCGGATGGCG (n=2)	-17,+7
					CCCGAGTGGAACGGTACGGCGGTTAGCGACGATGCCAA-----------ccgcgcctgtTAGCGGATGGCG (n=2)	-11, +10
	TC-540, 549, 551, 560	1-3	**CFP^{-}/*cd*$^{-}$**	**B/*cd*$^{\Delta 11}$**	CCCGAGTGGAACGGTACG-------------------------CCGCCATAGCGGATGGCG (n=4)	-25
	TC-541,561	1-3	**CFP^{-}/*cd*$^{-}$**	**B/*cd*$^{\Delta 11}$**	CCCGAGTGGAACGGTA---------------------------CCGCCATAGCGGATGGCG (n=2)	-27†
	TC-542, 546, 553, 554, 558	1-3	**CFP^{-}/*cd*$^{-}$**	**B/*cd*$^{\Delta 11}$**	CCCGAGTGGAACGG----------------------------------cgctATAGCGGATGGCG (n=5)	-34,+4
	TC-543, 544, 550, 562, 563, 567	1-3	**CFP^{-}/*cd*$^{-}$**	**B/*cd*$^{\Delta 11}$**	CCCGAGTGGAACGGTACGGCGGTTAGCGACGAT-----GGCGGCCGCCATAGCGGATGGCG (n=6)	-5†
	TC-545, 547, 564, 568 **(OC-1169)**	1-3	**CFP^{-}/*cd*$^{-}$**	**B/*cd*$^{\Delta 11}$**	CCCGAGTGGAACGGTACGGCGGTTAGCGAC--------------------------------- (n=4)	-83
	TC-552, 559, 565	1-3	**CFP^{-}/*cd*$^{-}$**	**B/*cd*$^{\Delta 11}$**	CCCGAGTGGAACGGTACGGCGGTTAGCGACGATGCC-------------tgtTAGCGGATGGCG (n=3)	-13,+3
	TC-556, 557, 566	1-3	**CFP^{-}/*cd*$^{-}$**	**B/*cd*$^{\Delta 11}$**	CCCGAGTGGAACGGTACGGCGGTTAGCGACGAT---cgAAGGCGGCCGCCATAGCGGATGGCG (n=3)	-3,+2
	TC-555	1-3	**CFP^{-}/*cd*$^{-}$**	**B/*cd*$^{\Delta 11}$**	CCCGAGTGGAACGGTACGGCGGTTAGCGACGATGC--gctatggcggttgAGGCGGCCGCCATAGCGGATGGCG (n=1)	-2,+13 k
**F1 CFP^{+}/*cd*$^{-}$ ♀ X *Agcd*$^{\Delta 11}$ ♂**	TC-408 **(OC-1141)**	1-3	**CFP^{-}/*cd*$^{+}$**	**B/*cd*$^{\Delta 11}$**	CCCGAGTGGAACGGTACGGCGGTTAGCGACGAT---tagcggcggttagcgctAAGGCGGCCGCCATAGCGGATGGCG (n=1)	-3,+17
	TC-569, 575, 581, 588, 592, 597	1-3	**CFP^{-}/*cd*$^{-}$**	**B/B/*cd*$^{\Delta 11}$**	CCCGAGTGGAACGGTACGGCGGTTAGCGACGATG--------gccGCCGCCATAGCGGATGGCG (n=6)	-8,+3
					CCCGAGTGGAACGGTACGGCGGTTAG-----------------CCGCCATAGCGGATGGCG (n=6)	-17†
	TC-570, 572, 579 **(OC-1161 and OC-1146)**	1-3	**CFP^{-}/*cd*$^{-}$**	**B/*cd*$^{\Delta 11}$**	CCCGAGTGGAACGGTAC---------------------GGCGGCCGCCATAGCGGATGGCG (n=3)	-21†
	TC-571, 573, 576, 578, 582, 583, 589, 591 **(OC-1152 and OC-1170)**	1-3	**CFP^{-}/*cd*$^{-}$**	**B/*cd*$^{\Delta 11}$**	CCCGAGTGGAACGGTACGGCGGTTAGCGA-----------CGGCCGCCATAGCGGATGGCG (n=8)	-11 g†
	TC-574, 580, 585, 586, 587, 594, 598 **(OC-1157)**	1-3	**CFP^{-}/*cd*$^{-}$**	**B/*cd*$^{\Delta 11}$**	CCCGAGTGGAACGGTACGGCGGTTAGCGACGATGCCA------------tgtTAGCGGATGGCG (n=7)	-12,+3
	TC-577, 584, 590, 593, 595, 596 **(OC-1159)**	1-3	**CFP^{-}/*cd*$^{-}$**	**B/*cd*$^{\Delta 11}$**	CCCGAGTGGAACGGTACGGCGGTTAGCGACGATGCCAcgatgccttAGGCGGCCGCCATAGCGGATGGCG (n=6)	+9 (stop)

[1]Wild-type and *cd*$^{\Delta 11}$ sequences are not listed.
[2]n is the number of that genotype sequenced.
[3]Mutation type: negative numbers are deletions; positive numbers are insertions; g is an identical genotype in multiple samples of F2 testcross progeny also seen in multiple F1 outcross progeny; i and j are identical genotypes in multiple samples of F2 testcross progeny seen only once in F1 outcross progeny; k is an identical genotype seen in multiple samples of F2 testcross progeny; † are sequences with microhomology end-joining features of 2-5-base-pair direct repeats in the canonical wild-type sequence.
Underlining indicates direct or inverted sequence insertions identical to a nearby genomic sequence.
Green shading highlights genotypes that also were found in outcross samples (OC sample identity in bold in parentheses).
Magenta highlighting signifies a possible source of an inherited allele from an OC individual (Table S2).
Grey highlighting signifies an inferred inherited allele based on the specific cross.
Abbreviations: Rep, replicate number; Phen, phenotype; Gen, genotype; CFP^{+} cyan fluorescent protein positive; *cd*$^{-}$, mutant *cardinal* gene; *cd*$^{+}$, wild-type *cardinal* gene; D, drive allele; R, functional mutant allele; B, non-functional mutant allele; W, wild-type allele. Sequence font colors represent the gRNA target, the PAM site and deletion or insertion.

Table S7. Lineage-dependent sources of independently-arising mutations in the testcross F2 progeny.			
**Outcross lineage**	**Testcross lineage**	**Representative sample[1]**	**Total**
Male	Male	TC-661, TC-746, TC-755	3
	Female	TC-747, TC-750	2
Female	Male	TC-281, TC-318, TC-421, TC-422, TC-482, TC-599, TC-601, TC-610, TC-612, TC-615	10
	Female	TC-427, TC-463, TC-471, TC-506, TC-511, TC-621, TC-629, TC-632, TC-634	9

[1]Includes only one entry from samples found in clusters.
Data from Tables S5 and S6.

Table S8. Cluster impacts on inheritance of R and B alleles in the male and female lineage testcrosses.

Parental cross X *Agcd*$^{\Delta 11}$ ♂ or ♀[1]	Allele[2]	Sample[3]	Cluster sizes[4]	Total alleles in cross[5]
**F1 parents from homozygous AgTP13 male outcrosses**				
**F1 CFP$^+$/*cd*$^+$ ♂**	**R**	TC-661 **(OC-1084 and OC-1348)**	**1**	**1**
**F1 CFP$^+$/*cd*$^+$ ♂**	**B**	TC-746 **(OC-1152 and OC-1170)**	**1**	**1**
**F1 CFP$^+$/*cd*$^+$ ♀**	**B**	TC-747-749 **(OC-1156)**	**3**	**5**
		TC-750-751	**2**	
**F1 CFP$^+$/Tear ♂**	**B**	TC-755-757	**3**	**3**
		**Total distinct mutations[6]**	**5**	
		**Total alleles scored[7]**		**10**
**F1 parents from homozygous AgTP13 female outcrosses**				
**F1 CFP$^+$/*cd*$^+$ ♂**	**R**	TC-281-300 (**OC-945 and OC-1369**)	**20**	**59**
		TC-301-317 **(OC-945 and OC-1369)**	**17**	
		TC-318-339 **(OC-929 and OC-1411)**	**22**	
	**B**	TC-479-481, 483-498, 500-505 **(OC-945 and OC-1369)**	**25**	**27**
		TC-482, 499	**2**	
**F1 CFP$^+$/*cd*$^+$ ♀**	**R**	TC-349, 351 **(OC-978)**	**2**	**33**
		TC-362, 365, 366, 371, 373, 377-380, 647-660 **(OC-1154 and OC-1392)**	**23**	
		TC-382, 383, 387, 388, 390, 392, 395, 399 **(OC-929 and OC-1411)**	**8**	
	**B**	TC-506-510 **(OC-1156)**	**5**	**33**
		TC-511-538	**28**	
**F1 CFP$^+$/Tear ♂**	**R**	TC-421	**1**	**5**
		TC-422-425	**4**	
	**B**	TC-601, 609, 611	**7**	**22**
		TC-610 **(OC-1156)**	**1**	
		TC-599-603	**5**	
		TC-612, 613, 614, 618, 619, 620 **(OC-945)**	**6**	
		TC-615-617	**3**	
**F1 CFP$^+$/ Tear ♀**	**R**	TC-426, 428, 432, 433, 436, 438, 440, 441, 444, 445, 450, 451, 453, 454 **(OC-1084)** and **OC-1348)**	**14**	**36**
		TC-427, 429, 430, 442, 443, 446, 447, 448, 452 **(OC-929 and OC-1411)**	**9**	
		TC-463, 464, 466, 467, 468, 470, 476, 477, 644-646 **(OC-945 and OC-1369)**	**11**	
		TC-471, 473 **(OC-929 and OC-1411)**	**2**	
	**B**	TC-621-628	**8**	**23**
		TC-629 **(OC-1152 and OC-1170)**	**1**	
		TC-630, 631, 633, 637, 638 **(OC-1121 and OC-1160)**	**5**	
		TC-632, 635, 639, 641, 642	**5**	
		TC-634, 636, 640, 643 **(OC-1152 and OC-1170)**	**4**	
**F1 CFP$^+$/*cd*$^-$ ♂**	**B**	TC-539, 548	**2**	**32**
			**2**	
		TC-540, 549, 551, 560	**4**	
		TC-541,561	**2**	
		TC-542, 546, 553, 554, 558	**5**	
		TC-543, 544, 550, 562, 563, 567	**6**	
		TC-545, 547, 564, 568 **(OC-1169)**	**4**	
		TC-552, 559, 565	**3**	
		TC-556, 557, 566	**3**	

		TC-555	**1**	
**F1 CFP^{+}/*cd*$^{-}$ ♀**	**R**	TC-401, 403, 404, 405, 407, 409, 410, 411, 412, 414, 419, 420	**12**	**19**
		TC-402, 413, 415-418	**6**	
		TC-406	**1**	
	**B**	TC-408 **(OC-1141)**	**1**	**37**
		TC-569, 575, 581, 588, 592, 597	**6**	
			**6**	
		TC-570, 572, 579 **(OC-1161** and **OC-1146)**	**3**	
		TC-571, 573, 576, 578, 582, 583, 589, 591 **(OC-1152 and OC-1170)**	**8**	
		TC-574, 580, 585, 586, 587, 594, 598 **(OC-1157)**	**7**	
		TC-577, 584, 590, 593, 595, 596 **(OC-1159)**	**6**	
		**Total distinct mutations[6]**	**46**	
		**Total alleles scored[7]**		**326**

[1]Drive-carrying parent crossed with *Agcd*$^{\Delta 11}$ males (♂) or females (♀).
[2]Alleles are functional (R) or non-functional (B) mutations of the *Agcd* gene.
[3]Samples are derived from Tables S5 and S6.
[4]R and B cluster sizes in the testcrosses are derived from Tables S5 and S6.
[5]Total R or B mutant alleles (singletons and clusters) in all replicates of the specific crosses derived from Tables S5 and S6.
[6]Total distinct mutations identified derived from Tables S5 and S6.
[7]Total alleles scored derived from Tables S5 and S6.
Magenta highlighting signifies a possible source of an inherited allele from an OC individual (Table S2).
Grey highlighting signifies an inferred inherited allele based on the specific cross.

Figure S1

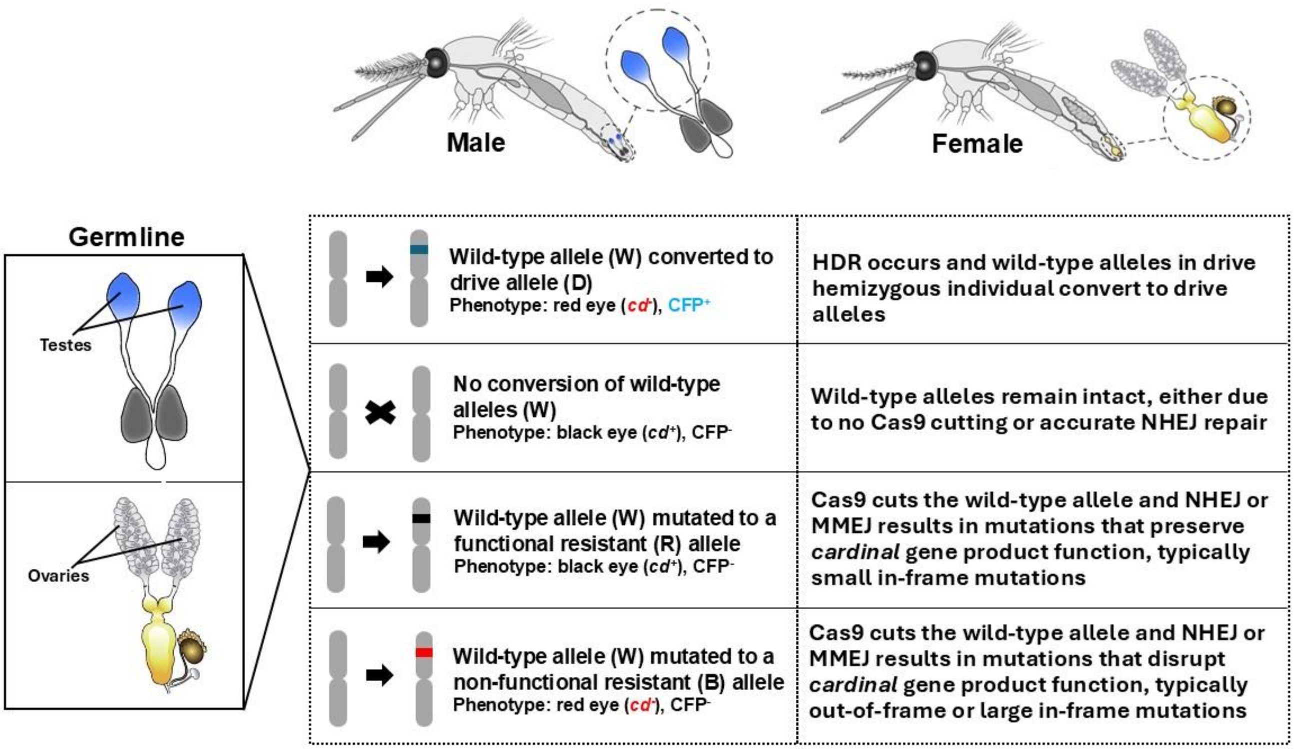

Figure S1. Outcross phenotype and genotype descriptions. The four outcomes of AgTP13 gene-drive activity in the male and female mosquito germlines. The same outcomes occur as a result of paternal and maternal effects. Abbreviations: CFP^{+} cyan fluorescent protein positive; CFP^{-} cyan fluorescent protein negative. Testes images adapted from Meuti and Short (2019) and mosquito outline and ovaries from Rogers *et al*. (2008; copyright [2008] National Academy of Sciences, U.S.A.), are used by permission.

Figure S2

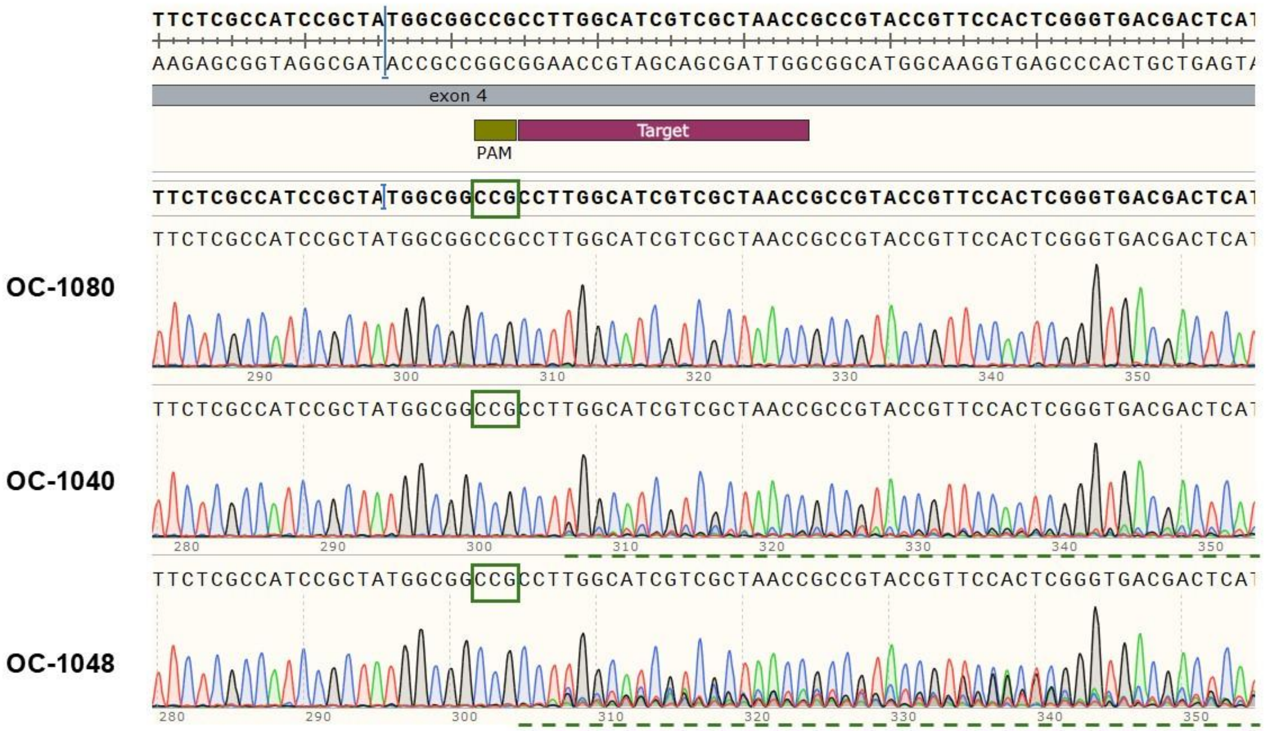

Figure S2. Examples of sequence tracings of cryptic and disturbed mosaic mosquitoes. OC-1080 is an example of a 'cryptic' trace. The traces are clean and the sequencing result shows only the reference wild-type sequence despite the sample coming from a mosquito with the tear (mosaic) phenotype indicative of the presence of a mutant allele, probably in small clone of cells. OC-1040 and OC1048 are example of a 'disturbed' (underlined) trace.

Figure S3

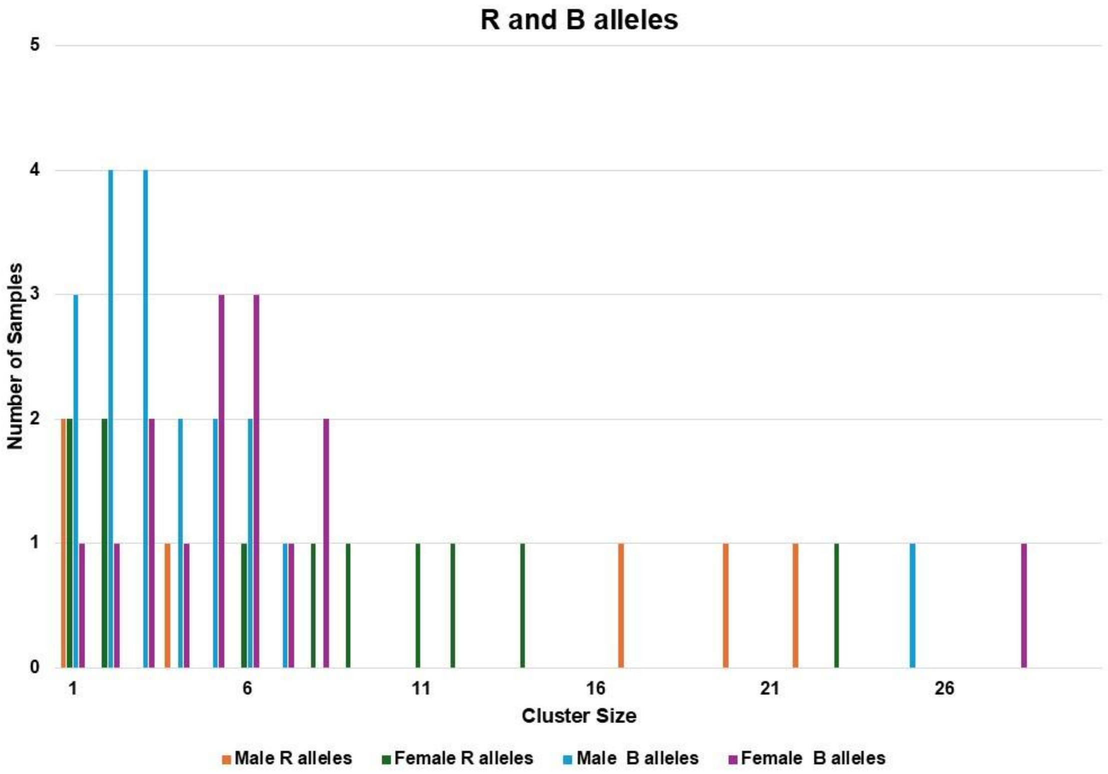

Figure S3. Distribution of R and B cluster sizes in F2 mosquitoes. The distribution of R and B alleles in males and females from all testcross samples.

References

Carballar-Lejarazú R, Ogaugwu C, Tushar T, Kelsey A, Pham TB, Murphy J, Schmidt H, Lee Y, Lanzaro GC, James AA. Next-generation gene drive for population modification of the malaria vector mosquito, *Anopheles gambiae*. PROC NATL ACAD SCI U S A. 2020 Sep 15;117(37):22805-22814. doi: 10.1073/pnas.2010214117. Epub 2020 Aug 24. PMID: 32839345; PMCID: PMC7502704.

Fiil A. 1976. Oogenesis in the malaria Mosquito Anopheles gambiae. CELL TISSUE RES. 167(1):23-35. doi: 10.1007/BF00220157.

Juhn J, James AA. 2012. Hybridization in situ of salivary glands, ovaries, and embryos of vector mosquitoes. J VIS EXP. (64):3709. doi: 10.3791/3709.

Meuti, M.E., Short, S.M. Physiological and Environmental Factors Affecting the Composition of the Ejaculate in Mosquitoes and Other Insects. INSECTS. 2019 Mar 15;10(3):74. doi: 10.3390/insects10030074. PMID: 30875967; PMCID: PMC6468485.

Nicholson, AJ. 1921. The development of the ovary and ovarian egg of a mosquito, *Anopheles maeutipennis,* J CELL. SCI. 65(259), 395-448. doi.org/10.1242/jcs.s2-65.259.395.

Rogers, D.W., Whitten, M.M., Thailayil, J., Soichot, J., Levashina E.A., Catteruccia, F. Molecular and cellular components of the mating machinery in Anopheles gambiae females. PROC NATL ACAD SCI U S A. 2008 Dec 9;105(49):19390-5. doi: 10.1073/pnas.0809723105. Epub 2008 Nov 26. PMID: 19036921; PMCID: PMC2614771.

Terradas G, Hermann A, James AA, McGinnis W, Bier E. 2022. High-resolution in situ analysis of Cas9 germline transcript distributions in gene-drive Anopheles mosquitoes. G3 (BETHESDA) 12(1):jkab369. doi: 10.1093/g3journal/jkab369.

Vitale M, Liang J, Sharakhov I, Bernardini F. 2023. Whole-Mount Fluorescence In Situ Hybridization to Study Spermatogenesis in the Anopheles Mosquito. J VIS EXP. 195. doi: 10.3791/65356.
